# Policies and processes for human papillomavirus vaccination in Latin America and the Caribbean

**DOI:** 10.26633/RPSP.2017.124

**Published:** 2017-12-20

**Authors:** Fernando De la Hoz Restrepo, Nelson Alvis Guzman, Alejandro De la Hoz Gomez, Cuauhtémoc Ruiz

**Affiliations:** 1 Universidad Nacional de Colombia Facultad de Medicina Bogotá Colombia Universidad Nacional de Colombia, Facultad de Medicina, Bogotá, Colombia.; 2 Universidad de Cartagena Facultad de Economía Bolívar Colombia Universidad de Cartagena, Facultad de Economía, Bolívar, Colombia.; 3 Pontificia Universidad Javeriana Facultad de Medicina Bogotá Colombia Pontificia Universidad Javeriana, Facultad de Medicina, Bogotá, Colombia.; 4 Organización Panamericana de la Salud Unidad de Inmunización Integral de la Familia Washington, D.C. United States of America Organización Panamericana de la Salud, Unidad de Inmunización Integral de la Familia, Washington, D.C., United States of America.

**Keywords:** Papillomavirus vaccines, health policy, health services research, program evaluation, immunization programs, West Indies, Latin America, Vacunas contra papilomavirus, política de salud, investigación en servicios de salud, evaluación de programas y proyectos de salud, programas de inmunización, Indias Occidentales, América Latina, Vacinas contra papilomavirus, política de saúde, pesquisa sobre serviços de saúde, avaliação de programas e projetos de saúde, programas de imunização, Índias Ocidentais, América Latina

## Abstract

**Objectives.:**

Three highly effective vaccines are available to prevent human papillomavirus (HPV) infection, and they have been introduced in many countries around the world. This article describes advances and challenges in introducing HPV vaccines in the Expanded Program of Immunization (EPI) of countries in Latin America and the Caribbean (LAC).

**Methods.:**

We reviewed national and regional sources of information to identify LAC countries with and without universal HPV vaccination, along with the year of introduction, type of HPV vaccine, vaccination scheme, age groups targeted, and coverage level reached. Incidence rates of cervical cancer were compared across countries with and without an HPV vaccination program, in order to identify inequities in access to HPV vaccines.

**Results.:**

So far, 10 LAC countries have supplied data on their vaccination policies and vaccination coverage rates to the Pan America Health Organization. The majority of those 10 started their vaccination programs using quadrivalent vaccine. Only Chile, Ecuador, and Mexico started their programs using a two-dose scheme. However, by the end of 2016, most of the other countries had switched from a three-dose to a two-dose scheme. Different age groups are targeted in the various programs. Some countries vaccinate one-year birth cohorts, while others vaccinate multiple-year birth cohorts. By the end of 2014, coverage with at least two doses ranged from a low of 2% to a high of 86%. With the exception of Venezuela, the LAC countries with the largest populations introduced universal HPV vaccination between 2010 and 2014. Despite the progress that has occurred in some LAC countries, there are still 10 LAC nations with cervical cancer rates above the LAC average (21.2 cases per 100 000) that have not introduced an HPV vaccine in their EPI.

**Conclusions.:**

With several key adjustments, HPV vaccination programs across Latin America and the Caribbean could be substantially strengthened. Ongoing monitoring of HPV infection outcomes is needed in order to assess the impact of different vaccination policies.

## RESUMEN

**Objetivos.:**

Existen tres vacunas de gran eficacia para prevenir la infección por el virus del papiloma humano (VPH) que se han introducido en muchos países en todo el mundo. En el presente artículo se describen los progresos alcanzados y las dificultades encontradas con la introducción de las vacunas contra el VPH en el Programa Ampliado de Inmunización de los países de América Latina y el Caribe.

**Métodos.:**

Se examinaron las fuentes de información nacionales y regionales con el fin de definir los países de América Latina y el Caribe que cuentan con la vacunación universal contra el VPH o que carecen de ella, además del año de introducción, el tipo de vacuna utilizada, el calendario de vacunación, los grupos de edad destinatarios y el grado de cobertura alcanzado. Se compararon las tasas de incidencia del cáncer cervicouterino entre los países que poseen un programa de vacunación contra el VPH y los países que no lo tienen, con el propósito de detectar las inequidades en el acceso a estas vacunas.

**Resultados.:**

Hasta el momento, diez países de América Latina y el Caribe han comunicado a la Organización Panamericana de la Salud los datos sobre sus políticas de vacunación y las tasas de cobertura. La mayoría de estos países comenzó sus programas de vacunación utilizando la vacuna tetravalente. Solo Chile, Ecuador y México iniciaron su programa con la aplicación de un esquema de dos dosis. Sin embargo, a fines del 2016 la mayoría de los demás países había pasado de un calendario de tres dosis a una pauta de dos dosis. Los programas se dirigen a diferentes grupos de edad. Algunos países vacunan cohortes de nacimiento de un año y otros vacunan cohortes de nacimiento de varios años. A fines del 2014, la cobertura con al menos dos dosis oscilaba entre un 2% y un 86%. Con la excepción de Venezuela, los países de América Latina y el Caribe con poblaciones más grandes introdujeron la vacunación universal contra el VPH del 2010 al 2014. Pese a los progresos alcanzados en algunos países existen todavía diez naciones en América Latina y el Caribe, cuyas tasas de cáncer cervicouterino son superiores al promedio de la Región (21,2 casos por 100 000 habitantes), que no han integrado la vacuna contra el VPH en su Programa Ampliado de Inmunización.

**Conclusiones.:**

Será necesario adoptar varias modificaciones esenciales con miras a fortalecer de manera sólida los programas de vacunación contra el VPH en América Latina y el Caribe. Es preciso llevar a cabo un seguimiento continuo de los resultados en materia de infección por el VPH que permita evaluar la repercusión de las diferentes políticas de vacunación.

## RESUMO

**Objetivos.:**

Existem três vacinas bastante eficazes para prevenir a infecção pelo papilomavírus humano (HPV) que foram introduzidas em diversos países em todo o mundo. Este artigo descreve os avanços e os desafios para introduzir as vacinas contra HPV no Programa Ampliado de Imunização (PAI) dos países na AméricaLatina e Caribe.

**Métodos.:**

Foram pesquisadas fontes de informação nacionais e regionais para identificar os países da América Latina e Caribe com e sem um programa de vacinação universal para HPV, assim como ano de introdução da vacina, tipo de vacina usada, esquema de vacinação, faixas etárias cobertas e nível de cobertura alcançado. Foram comparadas as taxas de incidência do câncer do colo uterino entre os países com e sem programa de vacinação a fim de identificar desigualdades de acesso à vacina contra HPV.

**Resultados.:**

Até o presente, 10 países da América Latina e Caribe forneceram dados sobre suas políticas de vacinação e taxas de cobertura de vacinação à Organização Pan-Americana da Saúde. A maioria iniciou introduzindo no programa de vacinação a vacina quadrivalente. Somente o Chile, Equador e México começaram seus programas com um esquema de duas doses. Porém, ao final de 2016, grande parte dos demais países havia migrado de um esquema de três para duas doses. As faixas etárias cobertas variam nos diversos programas: alguns países vacinam coortes de crianças com um ano de idade, outros vacinam coortes de crianças com idades variadas. Ao final de 2014, a cobertura com a aplicação de pelo menos duas doses da vacina variou de um nível bem baixo de 2% a um nível elevado de 86%. À exceção da Venezuela, os países mais populosos da América Latina e Caribe introduziram a vacinação universal para HPV entre 2010 e 2014. Apesar do progresso verificado, 10 nações onde foram registradas taxas de incidência de câncer do colo uterino acima da média da região (21,2 casos por 100.000 habitantes) ainda não introduziram a vacina contra HPV no próprio PAI.

**Conclusões.:**

Se forem tomadas várias medidas importantes, é possível reforçar consideravelmente os programas de vacinação para HPV na América Latina e Caribe. O monitoramento contínuo dos resultados se faz necessário para avaliar o impacto das políticas de vacinação

Infection with human papillomavirus (HPV) is a necessary–but not sufficient–cause for cervical cancer (1, 2). Despite advances in cervical cancer secondary prevention through the use of cervical cancer screening, there is still a substantial burden of disease due to HPV (3). Worldwide, it has been estimated that more than 500 000 cases and 250 000 deaths occur every year (1, 2). In Latin American and the Caribbean (LAC), more than 80 000 cases and 40 000 deaths occur every year, with more than 50% of the cases related to HPV types 16 or 18 (4).

Cervical cancer results from persistent infection with HPV. Though there are more than 100 HPV types, just two of them, HPV 16 and HPV 18, are responsible for more than 50% of the global burden of cervical cancer. Two highly efficacious vaccines that protect against HPV 16/18 persistent infection and highgrade lesions have been developed, and more than 57 countries around the world have introduced one of the vaccines. Recently, a 9-valent vaccine (Gardasil 9) was approved for human use, based on non-inferiority immunogenicity studies. It protects against carcinogenic types 31, 33, 45, 52, and 58, in addition to 16 and 18. The World Health Organization (WHO) recommends a schedule of two or three doses, depending on the age of the person to be vaccinated (4–6).

While most countries with universal HPV vaccination are in the developed world, some developing countries have also introduced universal HPV vaccination. In comparison to other developing regions, more countries in LAC have implemented government-funded universal HPV vaccination, and more women have been vaccinated (4, 7, 8). However, there is a paucity of information on how countries have undertaken this process and what the results have been so far (4, 9). The full impact that this intervention will have on the prevention of HPV-related cancers won’t be known until 20 to 30 years after the vaccine’s introduction. Nevertheless, there are several goals that can be monitored before cancer is reduced and that can be used as intermediate points for evaluation (6). One intermediate point to evaluate is the process of vaccine introduction, along with such intermediate outcomes as the coverage obtained with the vaccine, the age groups that have been covered, the type of vaccine selected by each country, and the vaccination scheme (10).

This article describes advances in several aspects of HPV vaccine introduction in LAC countries with government-funded HPV vaccination activities. It is hoped that the information provided here encourages LAC decisionmakers to strengthen the amount and quality of the resources devoted to preventing HPV-related diseases and to filling gaps in access to HPV vaccine.

## METHODS

This descriptive study focuses on the current state of government-funded HPV vaccination programs in a sample of LAC countries. This article also analyzes the presence of inequalities or inequities in the HPV vaccine introduction process in LAC nations, using a country-level ecological analysis of cervical cancer incidence rates among countries with or without universal HPV vaccination. The analysis was done to identify whether the nations in most need of the vaccine—ones with the highest incidence rates—have been offering the vaccine to women there.

The countries with government-funded HPV vaccination programs included in this analysis are: Argentina, Brazil, Chile, Colombia, Ecuador, Mexico, Panama, Paraguay, Peru, and Uruguay. They met the following two inclusion criteria: 1) having a government-funded HPV vaccination program, which means that vaccination is free for the target population, and 2) having information available on the number of doses applied since the beginning of the program. These 10 countries represent more than 70% of the population of LAC overall, and they report more than 60% of the cervical cancer cases occurring in LAC every year (11). With respect to Uruguay, the nation has a government-funded HPV vaccination program, but the country has not reported to the Pan American Health Organization (PAHO) the number of doses applied to its population. However, information on some other characteristics of the vaccination policies has appeared on the Web page of the Expanded Program on Immunization (EPI) of the Uruguayan Ministry of Health (MoH), so that material was included in this analysis.

Information on the HPV vaccination policies implemented in each of the selected countries was extracted from documents found on the Web page of the EPI of the respective ministry of health. Information on the type of vaccine, recommended scheme, and characteristics of the target population was extracted from the documents found on those Web pages. Information on the mnumber of doses applied was obtained from PAHO’s Comprehensive Family Immunization Unit, which conducts a survey every year asking LAC countries about the number of HPV vaccine doses delivered to target populations. That information is available stratified by age groups.

Population coverage by dose was esti-mated by age group and by country. The number of doses applied to each age group was divided by the estimated number of people within that age category. The number of persons in the denominator, by age group and country, was estimated using the population projections contained in the Demographic Bulletin produced by Economic Commission for Latin America and the Caribbean (CEPAL) in 2004 (12).

To assess the presence of inequalities in the HPV vaccine introduction process among LAC countries, the incidence of cervical cancer was described for the LAC countries with and without universal HPV vaccination by the end of the year 2014. The incidence of cervical cancer by country was obtained from the GLOBOCAN 2012 Web page (http://globocan.iarc.fr/Default.aspx). Incidence rates for countries with and without vaccination programs were compared against the overall LAC cervical cancer rate, which was 21.2 cases per 100 000 inhabitants in 2012.

## RESULTS

### Vaccination policies

Ten LAC countries fulfilled the selection criteria for being included in the analysis of HPV vaccination policies. Table 1 shows the main features of those countries’ policies (13–20). All countries started their vaccination programs targeting girls only, but recently Argentina, Brazil, and Panama started offering HPV vaccination to boys. Argentina, Ecuador, Panama, and Peru started the program using the bivalent vaccine but later switched to the quadrivalent form, except for Ecuador. Most countries have also changed vaccination schedules. With the exception of Chile, Ecuador, and Mexico, they started using a three dose scheme, although with different intervals between doses. Afterwards, many of them switched to a two-dose schedule, except for Colombia, Paraguay, and Uruguay.

There is wide heterogeneity in the target age selected for vaccination. Argentina, Panama, and Uruguay, have one-year birth cohorts (11, 10 , and 12 years old, respectively), while Brazil, Colombia, Ecuador, and Paraguay selected several-year birth cohorts (9–13 years, 9–17 years, 9–11 years, and 9–10 years, respectively). Mexico and Peru selected girls in the 5th grade of primary school.

**TABLE 1.** Public policies on HPV vaccination for selected Latin American and Caribbean countries with a government-funded HPV vaccination program

Country (ref.)	Target population	Vaccine	Vaccination schedule	Year introduced	Vaccine delivery
Argentina (13)	Girls 11 years old	Bivalent	0, 1, 6 months	2011	School based
		Quadrivalent	0, 2, 6 months	2014	School based
		Quadrivalent	0, 6 months	2015	School based
	Boys 11 years old	Quadrivalent	0, 6 months	2017	School based
Brazil (14)	Girls 9–13 years old	Quadrivalent	0, 6, 60 months	2014	School based
		Quadrivalent	0, 6 months	2016	School based
	Boys 12–13 years old	Quadrivalent	0, 6 months	2016	School based
Chile (15)	Girls 9 years old	Quadrivalent	0, 12 months	2014	School based
	Girls 10–14 years old	Quadrivalent	0, 12 months	2015	School based
Colombia	Girls 9–17 years old	Quadrivalent	0, 6, 60 months	2012	School based
Ecuador	Girls 9–11 years old	Bivalent	0, 6 months	2014	School based/health centers for girls out of school
Mexico	Girls in 5th grade of primary school/if not in school, 11 years old	Quadrivalent	0, 6 months	2012	School based/health centers for girls 11 yrs old who are out of school
Panama (16)	Girls 10 years old	Bivalent	0, 1, 6 months	2010	School based
		Quadrivalent	0, 6 months	2015	School based
	Boys 10 years old	Quadrivalent	0, 6 months	2016	School based
Paraguay	Girls 9–17 years old	Quadrivalent	0, 1, 6 months	2013	School based
Peru (17,18)	Girls in 5^th^ grade of primary school	Bivalent	0, 2, 6 months	2011	School based
		Quadrivalent		2016	School based
Uruguay^a^^,^ ^b^	Girls 12 years old	Quadrivalent	0, 2, 6 months	2013	Health center based

***Source:*** PAHO Comprehensive Family Immunization Unit database, plus documents from countries’ ministry of health Web pages.

a Need parent’s signed consent form.

b Since 2016, HPV vaccination is compulsory.

The vaccination strategy also differs among the countries. Most of them use only school-based vaccination, but Uruguay uses just health center–based vaccination. In addition, Uruguay asks for informed consent from a parent before an adolescent girl can be vaccinated.

Interestingly, Trinidad and Tobago introduced the vaccine for one year (2013) but subsequently suspended use of it.

### Vaccination coverage

Table 2 shows vaccination coverage by country, year, and age target group. Coverage with the first dose ranged from 9% to 98%. The highest coverage with the first dose was in Brazil for the first year of vaccine introduction, while the lowest occurred in Peru for the 2013 cohort (three years after vaccine introduction). The highest coverage for the first dose was in Ecuador (94%) and Panama (92%). Coverage for the first dose decreased with subsequent cohorts in Argentina, Brazil, Colombia, and Mexico.

A decline in coverage between the first and second dose was observed for most countries, with the exception of Mexico. Remarkably, Mexican 5th grade/11-year old girls had the same coverage with the first and second dose (86%). Other countries with high two-dose coverage were Panama, Paraguay, and Ecuador. In terms of coverage with three doses, Panama had the best coverage, at 75% for 2010–2014, while Peru had the lowest (3%) in 2014.

### Differences in cervical cancer incidence and access to HPV vaccination

Table 3 shows the incidence of cervical cancer (CC) among 12 LAC countries that had not introduced HPV vaccination by the end of 2015 (21–23). Except for Costa Rica and Cuba, these countries have incidence rates above the overall LAC average, which was 21.2 per 100 000 women in 2012.

The risk of cervical cancer is remarkably higher for Bolivia, Nicaragua, Venezuela, and the Dominican Republic. Compared to an average LAC woman, a woman living in one of those countries would have an increased risk of developing cervical cancer ranging from 44% to 125%. The incidence rate ratio (IRR), compared to the LAC average, is 2.25 for Bolivia, 1.71 for Nicaragua, 1.55 for Venezuela, and 1.45 for the Dominican Republic. Cuba and Costa Rica have a risk of cervical cancer that is lower than the LAC average, with an IRR of 0.81 for Cuba and 0.54 for Costa Rica.

Table 4 shows that 6 out of 10 countries with government-funded HPV vaccination programs had a lower incidence of cervical cancer than the LAC average. These 10 countries contribute 70% of the burden of disease caused by cervical cancer in LAC, more likely because of their demographic weight rather than due to an increased risk.

## DISCUSSION

According to Bruni et al. (8), 20 countries in the Americas have introduced publicly funded universal HPV vaccination. In this analysis, no data from Canada or the United States of America were analyzed because there is already a substantial amount of scientific literature on their HPV vaccination processes. Other countries of the Americas, as cited by Bruni et al., that have HPV vaccination programs and that were excluded from this analysis were: Bahamas, Barbados, Belize, Bermuda, Cayman Islands, Guyana, and Suriname. In addition, those authors identified Trinidad and Tobago and the Dominican Republic as countries with HPV vaccination, but the Dominican Republic only did that in 2017 (22), and Trinidad and Tobago suspended its vaccination program (Table 3).

**TABLE 2.** HPV vaccination coverage in selected countries of Latin America and the Caribbean

Country	Target population	Vaccine type	Vaccine schedule	Year(s)	Coverage		
					1st dose	2nd dose	3rd dose
Argentina	Girls 11 years old	Bivalent	0, 1, 6 months	2011	88%	59%	NA^a^
	Girls 11 years old	Bivalent	0, 1, 6 months	2012	68%	57%	38%
	Girls 11 years old	Bivalent	0, 1, 6 months	2013	79%	66%	51%
	Girls 11 years old	Quadrivalent	0, 2, 6 months	2014	67%	54%	43%
Brazil	Girls 11–13 years old	Quadrivalent	0, 6, 60 months	2014	98%	54%	*^b^
	Girls 9–10 years old	Quadrivalent	0, 6, 60 months	2015	73%	26%	*^b^
Chile	Girls 9 years old	Quadrivalent	0, 12 months	2014	81%	**^c^	NA
Colombia	Girls 9–17 years old	Quadrivalent	0, 6, 60 months	2012-2013	78%	40%	*^b^
	Girls 9 years old	Quadrivalent	0,6, 60 months	2014	37%	6%	*^b^
Ecuador	Girls 9–11 years old	Bivalent	0, 6 months	2014	94%	72%	NA
Mexico	Girls in 5th grade of primary school/11 years old	Quadrivalent	0, 6 months	2012-2013	86%	86%	NA
	Girls 14 years old	Quadrivalent	0, 6 months	2012	76.0%	4%	NA
Panama	Girls 10 years old	Bivalent	0, 1, 6 months	2010-2014	94%	84%	75%
Paraguay	Girls 9 years old	Quadrivalent	0, 1, 6 months	2013	79%	74%	71%
	Girls 9–10 years old	Quadrivalent	0, 1, 6 months	2014	80%	73%	50%
Peru	Girls in 5th grade of primary school	Bivalent	0, 2, 6 months	2011	58%	53%	33%
	Girls in 5th grade of primary school	Bivalent	0, 2, 6 months	2012	14%	13%	19%
	Girls in 5th grade of primary school	Bivalent	0, 2, 6 months	2013	9%	8%	8%
	Girls in 5th grade of primary school	Bivalent	0, 2, 6 months	2014	23%	2%	3%

***Source:*** PAHO Comprehensive Family Immunization Unit database, plus documents from countries’ ministry of health web pages.

a NA = not available.

b * = third dose had not been applied by the time this analysis was completed because the time interval between second and third dose was 5 years.

c ** = third dose had not been applied by the time this analysis was completed.

**TABLE 3.** Incidence of cervical cancer in 2012 for selected Latin American and Caribbean countries without an HPV vaccination program at end of 2014

Country (ref.)	Cervical cancer rate per 100 000	Cases (no.)	Deaths (no.)
Bolivia (21)	47.7	2 029	845
Nicaragua	36.2	934	424
Venezuela	32.8	4 973	1 789
Dominican Republic (22)	30.7	1 507	600
Hondurasa^a^ (23)	29.4	991	417
Jamaica	26.3	392	185
Haiti	24.9	1 048	575
El Salvador	24.8	823	388
Trinidad and Tobagob^b^	24.5	209	105
Guatemala	22.3	1 393	672
Cuba	17.1	1 287	569
Costa Rica	11.4	297	116
Total		15 883	6 685

***Source:*** GLOBOCAN 2012 (http://globocan.iarc.fr/Default.aspx).

a Since 2016, Honduras has had universal HPV vaccination among female adolescents.

b Trinidad and Tobago introduced a school-based HPV vaccination program for girls 11-13 years old in 2013, but it was suspended later that same year.

Reasons for exclusion from this analysis varied by country. Bermuda and Belize were excluded because they had not reported data to PAHO on coverage by the end of 2014. Guyana suspended HPV vaccination at some point but resumed it in 2016, according to a newspaper report (https://guyanachronicle.com/2016/10/22/hpv-vaccination-project-re-launched). Suriname reported to PAHO that 15 434 doses were applied in 2013 and that 15 593 were applied in 2014, but the country did not specify the age of the persons vaccinated.

The 10 LAC nations with government funded HPV vaccination program that were included in this analysis accounted for 70% of the disease burden caused by cervical cancer in LAC in 2012. This indicates the potential for a substantial decrease in HPV infections and related neoplasia in those 10 countries over time. Several key factors explain the relatively quick implementation of HPV vaccination in LAC countries. First, most LAC countries have a national immunization technical advisory group (NITAG) incorporated in their EPI structure, which provides support for technical discussions on introducing new vaccines (24). Second, the existence of the PAHO Revolving Fund gives nations in the Americas the ability to negotiate affordable prices for new vaccines, which facilitates the decision-making process (25). HPV vaccine prices for LAC countries have fallen from more than US$ 80.00 per dose to less than US$ 10.00 (26).

Nevertheless, challenges remain for completing the process of introducing HPV vaccination in LAC. First, there is striking inequality among LAC countries in the access to HPV vaccination. The nations with the highest rates of cervical cancer have been unable to introduce the vaccine. Second, there are gaps in coverage inside countries since many girls starting vaccination have been unable to complete the scheme. Third, there is an probably differ from country to country. In Colombia, a steep fall in coverage was related to a widely publicized outbreak of massive psychogenic illness attributed to HPV vaccination (34). In Uruguay, lower coverage has been explained by approaches used to identify and vaccinate thetarget population. The country is using a health center–based strategy for vaccination. In the first year after vaccination started, there was a requirement for a medical order and a signed consent from a parent or guardian of the girl for the vaccine to be applied. After evaluating the causes of low vaccination rates, Uruguay removed some of the barriers (20). In Peru, low coverage was at least partially related to finanurgent need to establish reliable and feasible surveillance systems on the prevalence of HPV infection and the prevalence of HPV-related precancerous cervical lesions. Fourth, there is a need to assess whether HPV vaccine introductions have been aligned with changes in the existing cervical cancer screening programs. Fifth, three countries (Argentina, Brazil, and Panama) have already introduced universal vaccination for boys, which may lead to faster control of HPV-related cancers and other lesions. However, that step may also increase inequalities in access to HPV vaccine among countries since a surge in vaccine demand may make it harder for the poorest countries to purchase it.

**TABLE 4.** Cervical cancer incidence in 2012 for selected Latin American and Caribbean countries with a government-funded HPV vaccination program as of the end of 2014

Country	Cervical cancer rates per 100 000	Cases (no.)	Deaths (no.)
Paraguay	34.2	1 022	439
Peru	32.7	4 636	1 715
Ecuador	29.0	2 094	1 026
Mexico	23.3	13 960	4 769
Argentina	20.9	4 956	2 127
Uruguay	19.0	402	175
Panama	18.7	351	134
Colombia	18.7	4 661	1 986
Brazil	16.3	18 503	8 414
Chile	12.8	1 441	734
Total		52 026	21 519

***Source:*** GLOBOCAN 2012 (http://globocan.iarc.fr/Default.aspx).

Cervical cancer occurrence can be a marker of health inequality (27). A similar pattern may now be emerging with HPV vaccination access. Of the 12 LAC countries that did not have an HPV vaccination program in 2015, 10 of them had a CC incidence rate above the LAC average (Table 3). Furthermore, those countries without an HPV vaccination program contribute substantially (about 23%) to the LAC cervical cancer burden. For most countries that have not introduced HPV vaccination, the reason has been financial. That is despite the fact that, at the current prices provided by the PAHO Revolving Fund, the intervention would be highly cost-effective for most of them (28). Two of them—Bolivia and Honduras—are applying for financial support from Gavi, the Vaccine Alliance, but the others are not eligible for that source of aid.

For countries that have not yet introduced the HPV vaccine, using a two-dose scheme would be advisable. Several studies have shown that a two-dose scheme would not reduce the effectiveness of the vaccine in preventing persistent infection. That approach would also encourage decisionmakers to prioritize investment in CC prevention (29, 30). Recently, WHO updated its recommendations for HPV vaccination, and is now encouraging countries to adopt a two-dose schedule (31).

In our research on the LAC nations, we observed striking differences in vaccination strategies among the countries. The nations could be influenced by the cost of HPV vaccination and differences in the age at first sexual intercourse. Vaccine cost may have affected the type of HPV vaccine used, the number of doses, and the time between doses. There are also differences in strategies designed to reach the target population. School-based vaccination is the method preferred in most countries, but some nations have relied more on vaccination at health centers. An analysis of the HPV vaccination policies in Europe also found important differences in HPV vaccination strategies among countries (32).

Current HPV vaccines used in LAC only cover carcinogenic types 16/18, which are responsible for about 50% of high-grade lesions and 60% to 65% of cervical cancers in LAC (10, 22). A recent analysis by Castellsagué et al. (33) showed that HPV16/18 vaccines would prevent 50% of cervical cancers in women 15 to 26 years old and 60% among women older than that. The 9-valent vaccine includes HPV types 31, 33, 45, 52, and 58, which are associated with 33% to 38% more CC in younger women and 17% to 18% more among older women. Despite its higher efficacy, 9-valent vaccine is not likely to be introduced soon in LAC, due to price barriers. As measured by coverage, most countries successfully applied the first dose of HPV, but coverage slumped for the second and third doses. Reasons for the decline cial restrictions. In 2015, the Government carried out a major effort to acquire more than 500 000 doses to complete unfinished schedules and vaccinate new cohorts (17).

### Conclusions

In summary, several adjustments should be made in order to bolster HPV vaccination programs across Latin America and the Caribbean. One pressing issue is unifying vaccination schemes. A two-dose scheme would substantially decrease financing pressures related to HPV vaccination, perhaps allowing more countries to introduce the vaccine. For countries that already have an HPV vaccination program, shifting to a two-dose scheme would release funds to ensure better follow-up on vaccination coverage and on analysis of vaccination barriers. In turn, that could help increase coverage. Another concern is adding better techniques to detect HPV in cervical samples in the existing cervical cancer screening programs in LAC countries, whether or not they are using an HPV vaccine. This would help control cervical cancer and decrease deaths from this cause.

#### Acknowledgments.

We are in debt with Ms Carmelita Pacis and Mr Raghunatan Gandhi who kindly provided us with the vaccine coverage database for this analysis

#### Funding.

This paper was partially supported by PAHO through a contract to Fernando De la Hoz

#### Disclaimer.

Authors hold sole responsibility for the views expressed in the manuscript, which may not necessarily reflect the opinion or policy of the RPSP/PAJPH or PAHO
